# Novel Angiotensin-Converting Enzyme Inhibitory Peptides from *Bungarus multicinctus*: Simulated Gastrointestinal Digestion, Identification and Antihypertensive Mechanism

**DOI:** 10.3390/ph19010096

**Published:** 2026-01-04

**Authors:** Yingying Ren, Han He, Yubin Cai, Shuyan Han, Ayzohra Ablat, Qiang Yin, Dandan Mu

**Affiliations:** 1 Key Laboratory of Xinjiang Phytomedicine Resource and Utilization, Pharmacy College of Shihezi University, Shihezi 832003, China; 20232115008@stu.shzu.edu.cn; 2 State Key Laboratory Basis of Xinjiang Indigenous Medicinal Plants Resource Utilization, Xinjiang Technical Institute of Physics and Chemistry, Chinese Academy of Sciences, Urumqi 830011, China

**Keywords:** *Bungarus multicinctus*, molecular docking, simulated gastrointestinal digestion, ACE inhibitory peptide, enzyme kinetics, stability analysis

## Abstract

**Background/Objectives**: Hypertension represents a leading contributor to cardiovascular disorders and premature mortality. Given the pervasive nature of adverse effects associated with current angiotensin-converting enzyme inhibitors (ACEIs), there is a significant interest in identifying novel bioactive lead compounds from natural sources. This study identifies, for the first time, three novel angiotensin-converting enzyme (ACE) inhibitory peptides released from *Bungarus multicinctus* (BM) via simulated gastrointestinal digestion (SGD). **Methods**: Active fractions were enriched by ultrafiltration and subjected to stability assessment. The peptide sequences were then determined using Liquid Chromatography-Tandem Mass Spectrometry (LC-MS/MS) and bioinformatics tools, followed by chemical synthesis. Finally, the inhibitory mechanism was investigated using kinetic analysis and molecular docking. **Results**: The intestinal digest exhibited potent ACE inhibition, with the <5 kDa fraction achieving 79% inhibition at 1 mg/mL and demonstrating favorable stability under varying temperatures, pH, and ionic strengths. Molecular docking revealed strong binding (affinity < −9.9 kcal/mol) of the peptides PPSPPRW, WGFTKF, and PSLFPPRL to key ACE residues—Tyr523, His513, and Arg522—via hydrogen and hydrophobic interactions. Enzyme kinetics characterized PPSPPRW and WGFTKF as competitive inhibitors, and PSLFPPRL as mixed type. The peptides demonstrated acceptable cell viability at lower concentrations, establishing a preliminary safety window for therapeutic application. **Conclusions:** These findings establish BM as a valuable source of stable, bioactive ACE-inhibitory peptides (ACEIPs) acting as promising lead compounds for antihypertensive therapies.

## 1. Introduction

Hypertension is a globally prevalent chronic cardiovascular disorder, affecting over 1.28 billion adults worldwide, and serves as a primary risk factor for cerebrovascular accident (CVA), myocardial infarction, and end-stage kidney disease—contributing to approximately 10.8 million premature deaths annually [1]. The renin-angiotensin system (RAS) serves as a central modulator of hemodynamic homeostasis, where the central enzyme ACE promotes vasoconstriction and hypertension. This is achieved through the conversion of angiotensin I to the potent vasoconstrictor angiotensin II, coupled with the degradation of the vasorelaxant peptide bradykinin [2]. Targeting ACE consequently emerged as a foundational therapeutic approach in controlling elevated blood pressure. Chemically synthesized ACE inhibitors, including lisinopril, enalapril, and captopril, represent the primary pharmaceutical option for managing hypertension. However, the chronic use of these drugs is linked to several adverse effects, such as a persistent non-productive cough (incidence: 5–20%), renal impairment, renal dysfunction, hyperkalemia, and angioedema [3,4], which limit patient compliance and clinical application. To address this limitation, the discovery of safe, effective, and naturally derived ACEIPs has emerged as a focus of pharmaceutical and medicinal chemistry research.

While numerous ACEIPs have been identified from diverse natural matrices, including soybeans, marine algae, and Morchella esculenta [5,6], their therapeutic translation is often constrained by modest inhibitory potency or limited oral bioavailability due to degradation by gastrointestinal proteases. This necessitates the continued exploration of novel sources for potent and stable peptide candidates. In this context, animal proteins are regarded as particularly valuable reservoirs for ACEIP discovery, owing to their comprehensive amino acid profiles and the structural diversity forged by distinct evolutionary pressures [7]. Among animal-derived sources, the proteomes of venomous fauna represent a frontier for peptide discovery. BM, recorded as “Jin Qian Bai Hua She” in the Pharmacopoeia of the People’s Republic of China, is a prominent traditional Chinese medicine. According to pharmacopoeial standards, the medicinal material is defined as the dried bodies of juvenile specimens. These specimens undergo rigorous processing involving visceral removal and specialized drying techniques to ensure quality and efficacy [8], the non-venom-derived proteins of this species remain a largely underutilized source for identifying bioactive peptides. This research gap is significant because these proteins are biochemically distinct from venom toxins and likely contain cryptic peptide sequences with novel therapeutic properties. In conclusion, our findings suggest that the unexploited protein biomass of *B. multicinctus* is a valuable resource for discovering novel ACEIPs with favorable potency, stability, and distinct mechanisms of action.

Many peptides identified through conventional in vitro hydrolysis, despite exhibiting potent initial activity, are limited in their practical efficacy due to their instability within the gastrointestinal environment. To overcome this bottleneck and select for peptides with both high activity and digestive stability, a standardized SGD model was employed prior to activity screening. This approach screens peptides with high stability, increasing the likelihood of their bioavailability and bioactivity

Therefore, applying an SGD model represents a crucial and logical step to unlock the true therapeutic potential of this unexplored proteome. As far as we are aware, this work represents the first to systematically investigate the release of ACEIPs from BM non-venom-derived proteins under conditions that mimic human digestion. To do this, we will first use a peptidomics-based approach to track how ACE inhibitory activity evolves and to pinpoint the key peptides released during a multi-stage simulated digestion process. Following their identification, we will then validate the potency of the most promising candidates by synthesizing them and exploring their inhibitory mechanisms through comprehensive kinetic and structural analyses. Ultimately, these findings are expected to not only reveal novel, digestion-stable ACEIPs but also provide a scientific foundation for repurposing BM as a valuable biomaterial for pharmaceuticals, expanding its use beyond traditional venom-focused research.

## 2. Results and Discussion

### 2.1. Characterization and ACE Inhibitory Activity of Bungarus Multicinctus Hydrolysates

**Figure 1:**
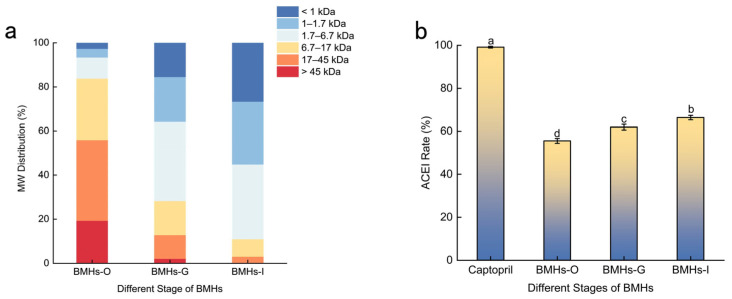
(**a**) Molecular weight distribution of BMHs at different digestion stages. (**b**) ACE inhibitory activity of BMHs from different stages of SGD. Captopril (1.0 mg/mL) was included as a positive control to validate the assay performance. Data are presented as mean ± SD (*n* = 3). Different letters (a–d) indicate statistically significant differences (*p* < 0.05).

Given that higher essential amino acid ratios and lower molecular weights (MW) confer superior bioavailability, the MW distribution, amino acid composition, and ACE inhibitory activity were compared across the three SGD stages, including the *Bungarus multicinctus* hydrolysates (BMHs) derived from the oral digestion phase (BMHs-O), the product of oral plus gastric digestion (BMHs-G), and the final product of oral, gastric, and intestinal digestion (BMHs-I), to identify a bioavailable, potent ACE-inhibitory hydrolysate for further study. As shown in Figure 1a, the average MW of BMHs decreased progressively throughout the SGD process. The intestinal-phase hydrolysates (BMHs-I) were predominantly composed of low-MW peptides (≤6.7 kDa), which accounted for approximately 81.10% of the total content. The abundance of these low-MW peptides is indicative of enhanced bioavailability, underscoring their potential functional and nutritional value [9].

Among the three BMHs studied, the BMHs-I exhibited significant ACE inhibitory activity (Figure 1b). The inhibition rate of BMHs-I was lower than that of the positive control, Captopril (99.2 ± 0.5%). This difference is likely because the hydrolysate is a mixture containing inactive substances, whereas Captopril is a pure compound. Importantly, the high activity of the control validates the performance of the assay. Furthermore, the activity of BMHs-I was markedly stronger than that of other animal protein hydrolysates; for instance, it was approximately 1.3 times more potent than earthworm hydrolysate (IC_50_ = 0.41 ± 0.11 mg/mL) [10] and substantially exceeded the inhibition rate of a tryptic casein hydrolysate, which was less than 30% at comparable concentrations [11]. The potent inhibitory effect of BMHs-I is consistent with its molecular weight profile, which was predominantly composed of low-MW peptides (Figure 1a). The high abundance of peptides within the reported optimal range for ACE inhibition (200–1800 Da) [12] supports the premise that the progression of SGD releases a greater number of bioactive peptides.

The amino acid composition of BMHs from different digestion phases is shown in Table 1. A notable trend was the progressive increase in essential amino acids (EAAs) throughout the digestion process, with contents of 19.78%, 28.6%, and 30.7% for BMHs-O, BMHs-G, and BMHs-I, However, this enrichment was not uniform across all residues. The rise in total EAA content was predominantly driven by the significant accumulation of hydrophobic branched-chain amino acids (BCAAs), specifically Val, Ile, and Leu. In contrast, residues such as Thr and Lys remained relatively stable, while Met showed a marked decline, likely due to oxidative degradation during processing. Meanwhile, Arg content continued to increase, implying selective cleavage patterns. Consequently, BMHs-I exhibited the highest total EAA content, characterized by a specific BCAA-rich profile rather than a global elevation of all essential amino acids. This profile closely aligns with the amino acid requirement pattern of the human body, indicating that BMHs-I possesses superior nutritional quality and an enhanced potential for protein absorption and utilization [13]. These characteristics underscore its promise as a high-value animal-derived ingredient.

### 2.2. Analysis of ACE Inhibitory Rates of BMHs-I Fractions with Different MW

**Figure 2:**
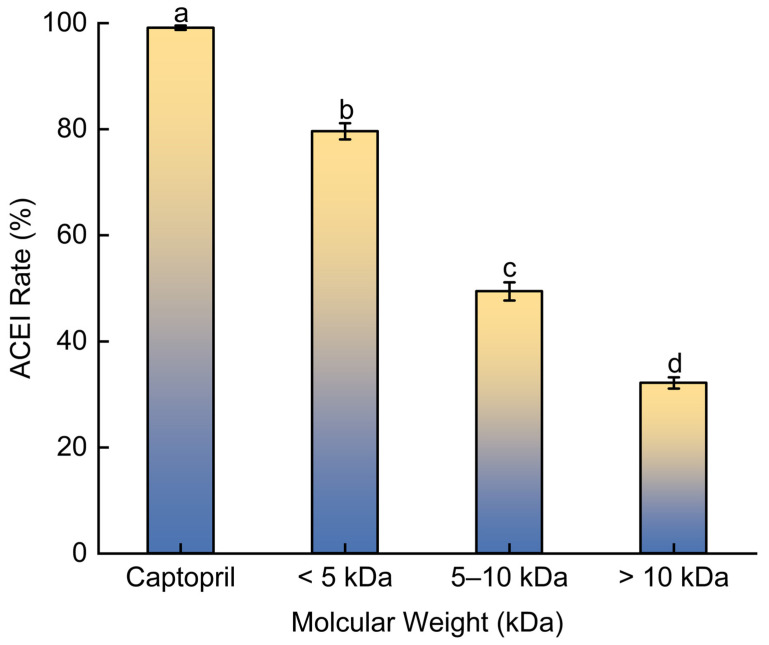
The rate of ACE inhibition by different fractions of BMHs-I. Captopril was included as a positive control to benchmark potency. Data are presented as mean ± SD (*n* = 3). Different letters (a–d) indicate statistically significant differences (*p* < 0.05).

Ultrafiltration was employed to fractionate the BMHs-I mixture based on molecular weight, aiming to enrich and concentrate the components with the highest ACE inhibitory activity (Figure 2). The <5 kDa fraction (designated as BMHs-IU) demonstrated the strongest inhibitory effect (79.1 ± 1.53%) when tested at 1.0 mg/mL, outperforming both the 5–10 kDa and >10 kDa components under evaluation. The positive control, Captopril validated the reliability of the assay system.

The BMH-I fraction (<5 kDa) exhibited higher ACE inhibition compared to the higher molecular weight fractions. While reduced steric hindrance allows smaller peptides to access the ACE active site more easily, molecular weight is not the sole determinant of potency. Inhibition efficacy is typically influenced by specific structural motifs, such as the presence of hydrophobic or aromatic residues, which are often found at the C-terminus. Thus, the observed activity of BMH-I likely results from the enrichment of peptides containing these favorable pharmacophores.

Moreover, its enhanced ACE inhibitory rate compared to previously reported (e.g., 75.88% inhibition by *Cyclina sinensis* [14]) hydrolysates underscores the abundance of antihypertensive peptides within this fraction, justifying its selection for further investigation.

### 2.3. Stability Analysis of BMHs-IU

Peptide structures are unstable and prone to degradation by certain factors, resulting in low bioavailability. Accordingly, the effects of temperature, pH, different NaCl concentrations, and metal ions on the stability of potential ACEI peptides in BMHs were investigated.

#### 2.3.1. Temperature Effects on BMHs-IU Component Stability

**Figure 3:**
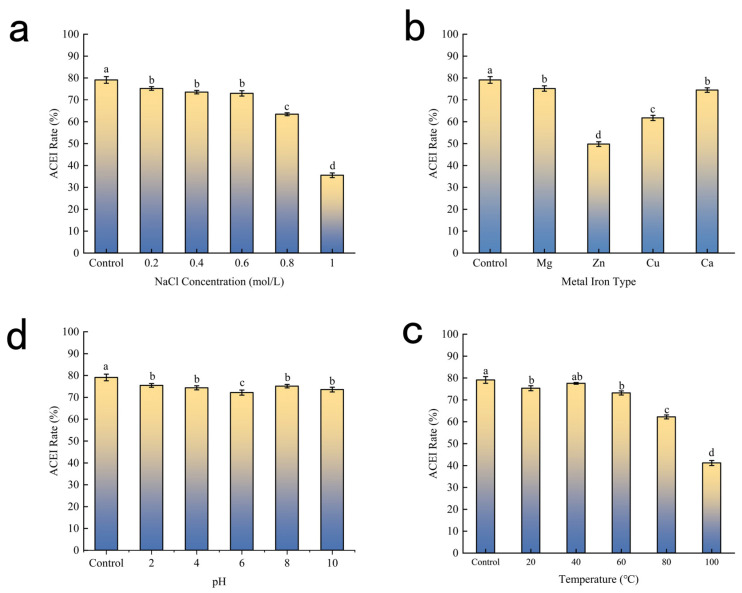
(**a**) Influence of Ionic Strength on the Stability of BMHs-IU; (**b**) Effects of Metal Ions on BMHs-IU Component Stability; (**c**) Temperature Effects on BMHs-IU Component Stability; (**d**) PH Effects on BMHs-IU Component Stability. Data are presented as mean ± SD (*n* = 3). Different letters (a–d) indicate statistically significant differences (*p* < 0.05).

The thermal stability profile of BMHs-IU is illustrated in Figure 3c. Relative to the positive control (79.10%), the ACE inhibitory activity of BMHs-IU exhibited stability up to 60 °C. Specifically, the peptide retained 73.16% ACE inhibitory activity at 60 °C, with no statistically significant difference from the control (*p* > 0.05). Conversely, thermal treatments exceeding this threshold precipitated a sharp attenuation in efficacy, with inhibition rates dropping to 62.22% at 80 °C and further declining to 41.16% at 100 °C (*p <* 0.05). This loss of function is likely driven by thermal denaturation and the subsequent unfolding of the peptide’s active secondary motifs. Given this thermal resilience, BMHs-IU demonstrates favorable properties for a peptide-based therapeutic candidate, suggesting it can withstand the thermal stresses often encountered during pharmaceutical processing and formulation [15].

#### 2.3.2. pH Effects on BMHs-IU Component Stability

The influence of pH on ACE inhibitory activity is presented in Figure 3d. To evaluate the peptide’s stability under varying acid-base conditions, the inhibitory rates across a pH spectrum (2–10) were compared against a Control maintained at neutral pH 7.0 (79.10%). The results indicate that BMHs-IU possesses robust stability against pH fluctuations. Compared to the Control, the peptide retained high inhibitory proficiency in both acidic and alkaline environments, with rates of 75.46% at pH 2 and 73.55% at pH 10. A slight, statistically non-significant reduction was observed at pH 6 (72.18%), likely because this value approximates the peptide’s isoelectric point [16], where protein structural contraction induces molecular aggregation, and where reduced net charge may induce minor aggregation. Overall, the maintenance of high activity (>72%) across the entire pH 2–10 range suggests that BMHs-IU is resistant to pH-induced conformational degradation.

#### 2.3.3. Influence of Ionic Strength on the Stability of BMHs-IU

The ACE inhibition rates under different ionic strengths are shown in Figure 3a. A Control group (0 mol/L NaCl) was utilized to determine the inhibitory rate in a pristine, salt-free environment. Compared to the Control, the addition of NaCl at low concentrations (0.2–0.6 mol/L) resulted in only a minor fluctuation in activity. However, a significant decline was observed when concentrations exceeded 0.6 mol/L. Mechanistically, this attenuation is likely driven by the dehydration of the peptide via the salting-out effect. As the hydration barrier is compromised, hydrophobic regions become exposed, leading to aggregation and the disruption of the bioactive conformation. Alternatively, high levels of Na^+^ may compete with the peptide for coordination with Zn^2+^ at the enzyme’s active site, steric hindrance that reduces binding affinity.

#### 2.3.4. Effects of Metal Ions on BMHs-IU Component Stability

Figure 3b illustrates the effects of different metal ions on the ACE inhibitory activity of the BMHs-IU peptide solution. Control represents the activity measured in a solution without any metal ions. Ca^2+^ and Mg^2+^ exerted the least influence, while Cu^2+^ and Zn^2+^, in contrast, exhibited more pronounced interference with the activity. This may be attributed to the fact that Zn^2+^ may bind to the active site of the peptide, markedly impairing its ACE inhibitory effect; Cu^2+^, on the other hand, may undergo coordination reactions with functional groups such as amino and carboxyl groups of the ACE inhibitory peptide, altering its spatial conformation and thereby either enhancing or reducing its inhibitory activity.

### 2.4. Characterization and Screening of Peptides

**Figure 4:**
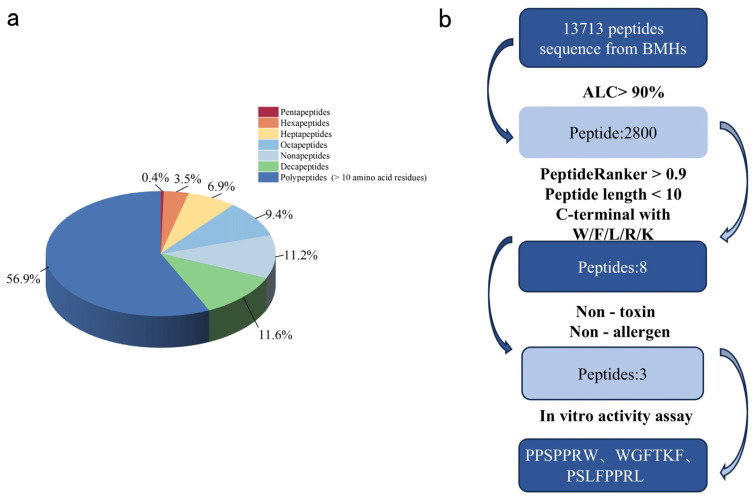
(**a**) Distribute the peptides identified in BMHs-IU by length. Sequences with 5–10 amino acids are labeled specifically, while sequences with more than 10 amino acid residues are categorized as polypeptides. (**b**) The process and outcomes of BMHs-IU screening using silico methods.

To identify the specific peptides mediating ACE inhibitory activity and improve ACEIP screening efficiency, LC-MS/MS was employed to characterize BMHs-IU (the fraction with the highest ACE inhibitory activity). A total of 13,713 peptides were identified in BMHs-IU. The length distribution analysis revealed a clear trend that the proportion of identified peptides generally increased with chain length, ranging from 0.4% for pentapeptides to 11.6% for decapeptides, with polypeptides (>10 residues) being the most abundant group at 56.9% (Figure 4a).

The number of peptide sequences identified from BMHs-IU was greater than that from plants and other animals—for example, over 3000 peptides were identified from tuna muscle [17] and 845 peptides from soybeans [5], while only 3160 peptides were identified from earthworm [10]. The substantially higher number of peptides identified in BMHs-IU, compared to other sources, likely reflects the complexity and diversity of the BM proteome. The distinctive protein composition of snake tissues may give rise to a broader array of unique peptide sequences upon enzymatic hydrolysis, underscoring the value of BM as a source for novel bioactive peptide discovery.

### 2.5. In Silico Screening of ACEIPs from BMHs-IU

The computer virtual screening process for ACEIPs is illustrated in Figure 4b. From an initial set of 13,713 peptides derived from BMHs-IU, a multi-step screening strategy was applied. Based on a stringent selection pipeline, 2800 peptides were initially identified with high confidence (Average Local Confidence, ALC > 90%) [18]. Subsequent screening using PeptideRanker (score > 0.9) yielded 22 candidate peptides. Subsequent filtering was applied based on two key criteria for ACE inhibitory potency: the presence of C-terminal aromatic or basic residues (W, F, R, K, L) [19] and a peptide length of fewer than nine amino acids [20]. This stepwise refinement produced a shortlist of eight candidate peptides. Final safety evaluation identified five non-toxic and non-allergenic peptides exhibiting the strongest predicted binding affinities, which were prioritized for chemical synthesis and experimental validation.

**Figure 5:**
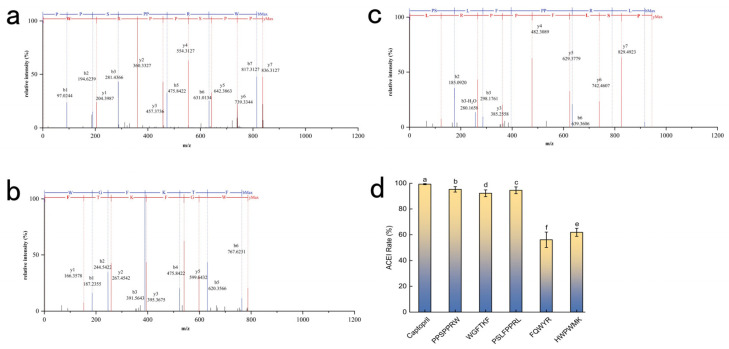
The second mass spectrometry spectrum of (**a**) PPSPPRW, (**b**) WGFTKF, (**c**) PSLFPPRL, (**d**) ACE inhibition by synthetic peptides (1.0 mg/mL). Captopril was included as a positive control to benchmark potency. Data are presented as mean ± SD (*n* = 3). Different letters (a–f) indicate statistically significant differences (*p* < 0.05).

The ACE inhibitory activities were validated at a concentration of 1.0 mg/mL (Figure 5d), corresponding to molar concentrations of 1.20 mM (PPSPPRW), 1.27 mM (WGFTKF), 1.25 mM (FQWYR), 1.13 mM (HWPWMK), and 1.08 mM (PSLFPPRL). While molecular docking provides insights into binding stability, the simulations were performed using energy-minimized static structures. However, in the actual reaction system, ACE does not necessarily bind to the peptide in the theoretically lowest-energy conformation. This discrepancy explains why peptides like FQWYR (−10.2 kcal/mol) and HWPWMK (−8.6 kcal/mol) (Table 2) displayed favorable binding energies but only moderate experimental activities. Captopril served as a reference to evaluate the inhibition level. The results indicated that the top candidates possessed significant inhibitory potential, justifying their selection for further analysis. Therefore, a strict selection threshold (>90% inhibition) was applied to prioritize experimental efficacy over theoretical prediction. Based on these findings, PPSPPRW, PSLFPPRL, and WGFTKF were identified as the key active components and were selected for further molecular interaction analysis (Figure 5a–c).

The BMHs-IU-derived peptides demonstrated higher activity than other animal-derived ACEIPs. At a concentration of 1 mg/mL, for instance, AEYLCEAC resultant from oyster protein hydrolysates, showed less than 20% ACE inhibition rate [21]. The low potency of AEYLCEAC (<20%) is likely attributable to disulfide bond-induced steric hindrance. Unlike the conformational stability provided by proline, this cross-linking may preclude the peptide from accessing the deep catalytic cleft of ACE. Furthermore, ADRYSSWP derived from earthworm showcased ACE inhibitory activity with 79.88% [10]. Overall, these findings not only confirm the presence of ACEIPs in BMHs-IU but also demonstrate the efficacy of an integrated peptidomics–bioinformatics approach for ACEIPs discovery.

### 2.6. Molecular Interaction Between Novel Peptides and ACE

**Figure 6:**
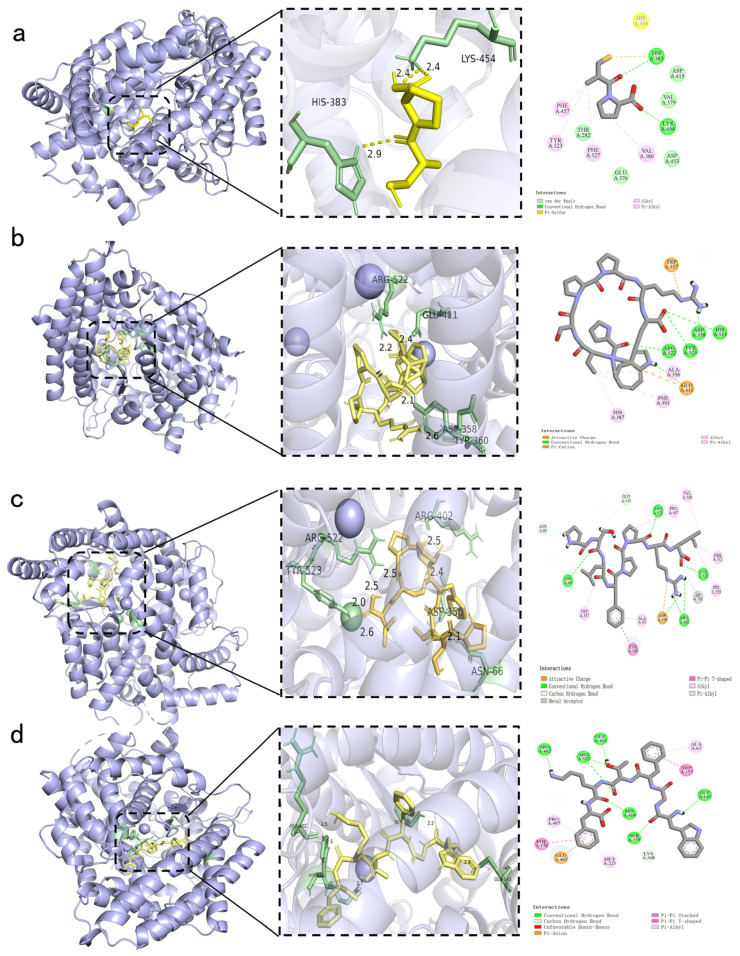
Molecular docking analysis of the positive control and identified peptides binding to the ACE active site. The figure displays the 3D binding conformations (**left**) and corresponding 2D interaction diagram (**right**) for: (**a**) the positive control Captopril; (**b**) PPSPPRW; (**c**) PSLFPPRL; and (**d**) WGFTKF. In the 3D visualizations, the ligand (peptide or Captopril) is represented by yellow sticks, while the key interacting amino acid residues within the ACE binding pocket are highlighted in green.

Molecular docking simulations predicted the effective binding of the positive control (Captopril) and three peptides, PPSPPRW, WGFTKF, and PSLFPPRL, to the active site of ACE (Figure 6a–d). The calculated binding energies were −11.1 kcal/mol for PPSPPRW, −10.4 kcal/mol for WGFTKF, and −9.9 kcal/mol for PSLFPPRL. These values were lower than those of Captopril (−5.5 kcal/mol), indicating stable binding. Furthermore, the binding affinity of these peptides was comparable to or more negative than those of other reported ACE-inhibitory peptides, such as FFFN (−9.6 kcal/mol) and FFDK (−8.6 kcal/mol) from *Torreya grandis* [22], and lower than those of peptides from *Hirudo nipponia* Whitman (−5.6~−7.2 kcal/mol) [23].

Molecular docking revealed distinct binding modes that are associated with inhibitory potency. PPSPPRW formed the most extensive network, occupying both S1 (Tyr523) and S2 (His513) pockets with four hydrogen bonds (Figure 5b). This orientation blocks substrate access to the catalytic center. Similarly, WGFTKF bound to the S1 pocket and formed hydrogen bonds with Arg522. PSLFPPRL also established a stable interaction with the active site, primarily driven by hydrophobic contacts and structural fit [24].

Notably, two key structural features elucidate the structure-activity relationship (SAR) and support the high potency (>90% inhibition) observed in our experiments. First, the hydrophobic C-termini of all three peptides insert deeply into the S1′ pocket, stabilizing the complex through hydrophobic or π-π stacking interactions—a critical requirement for ACE inhibition. Second, the N-terminal Proline residues (in PPSPPRW and PSLFPPRL) impart structural rigidity, reducing entropic penalty and facilitating a tight fit into the active site. These computational models provide a structural hypothesis that is consistent with the experimental inhibition data, confirming that specific active-site blockades and structural rigidity are the primary drivers of the inhibitory potency for these BMHs-IU-derived peptides. These structural observations are consistent with the functional ACE inhibition data and support the conclusion that both active-site blockade and auxiliary interactions beyond the catalytic pocket underpin the inhibitory potency of the identified BMHs-IU-derived peptides.

These structural observations are consistent with the functional ACE inhibition data and support the conclusion that both active-site blockade and auxiliary interactions beyond the catalytic pocket underpin the inhibitory potency of the identified BMHs-IU-derived peptides.

The ACE active site is located in a deep hydrophobic cleft containing a zinc ion (Zn^2+^) and specific pockets (S1 and S2) [25]. Analysis of the Captopril-ACE complex showed that it binds to key residues His383 and Tyr523. Consistent with this, the identified peptides also interacted with these residues. For example, the peptides targeted the S1 pocket anchored by Tyr523, occupying the same region as Captopril. However, unlike the small Captopril molecule, the peptides are larger and extend into auxiliary pockets, such as the S2 subsite. This allows them to block the active channel more effectively.

### 2.7. Enzyme Kinetic Analysis of Novel Peptides Against ACE

**Figure 7:**
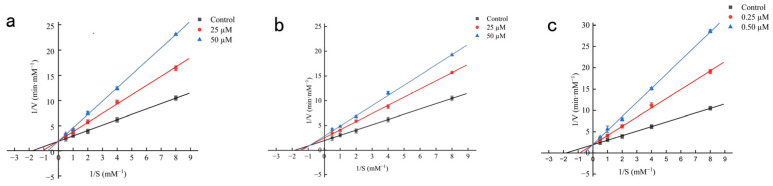
Lineweaver-Burk plots of synthetic ACEIPs from BMHs-IU. (**a**) Mode of inhibition by WGFTKF; (**b**) Mode of inhibition by PSLFPPRL; (**c**) Mode of inhibition by PPSPPRW.

Kinetic analyses delineated distinct inhibitory mechanisms of the three peptides toward ACE. Lineweaver–Burk plots showed that PPSPPRW and WGFTKF exhibited typical competitive inhibition, with lines converging at the y-axis, whereas PSLFPPRL displayed mixed-type inhibition, indicated by an intersection in the fourth quadrant (Figure 7). Consistent with these patterns, Michaelis–Menten kinetics revealed a concentration-dependent increase in K_m_ with unchanged V_max_ for PPSPPRW and WGFTKF, and a decrease in Vmax accompanied by increased Km for PSLFPPRL. These results collectively identify PPSPPRW and WGFTKF as competitive inhibitors. A similar inhibitory mode was also reported for the peptide (EASPKPV) isolated from ginkgo seed globulin [26] and the peptide (KWEKPF) isolated from the Chinese Rushan cheese by-product [26], both of which inhibited ACE activity competitively, and identified PSLFPPRL as a mixed-type inhibitor, analogous to green coffee bean peptide IIPNEVY [27].

Our findings reinforce the principle that the inhibitory behavior of peptides is dictated by their specific amino acid sequences and molecular interaction patterns [28]. The distinct mechanisms uncovered—competitive inhibition by PPSPPRW and WGFTKF versus mixed-type inhibition by PSLFPPRL—provide a mechanistic basis for the rational design of potent ACE-inhibitory peptides.

### 2.8. Cell Viability

**Figure 8:**
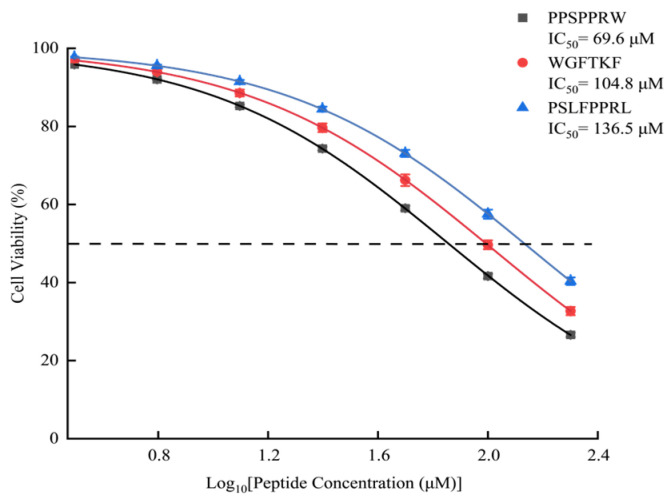
The effect of ACEIPs on HUVEC cell viability.

To determine the potential therapeutic window of the candidate peptides, a cytotoxicity assay was performed on HUVECs across a broad concentration range (Figure 8). Unlike food-derived peptides, which are often inert, these pharmacologically active peptides derived from *B. multicinctus* exhibited a dose-dependent influence on cell viability. The calculated IC50 values for cytotoxicity were 69.6 µM (PPSPPRW), 104.8 µM (WGFTKF), and 136.5 µM (PSLFPPRL). Notably, at lower concentrations (<25 µM), the cell viability remained above 90%, indicating a functional safety margin. As shown in Figure 8, similarly, WAGP (IC_50_ = 140.70 µM), a peptide derived from carnosine-containing protein, has been reported to exert antihypertensive effects.

These results suggest that while the peptides possess potent biological activity, their application as pharmaceutical agents requires careful dosage control. The observed cytotoxicity at higher concentrations (supra-physiological levels) underscores the nature of these peptides as potent lead compounds. This establishes a baseline for future medicinal chemistry efforts, such as structural modification or cyclization, aimed at improving the therapeutic index (widening the gap between the effective dose and the toxic dose) before progressing to in vivo applications.

The absence of cytotoxicity confirms that the ACE inhibition observed is a specific bioactivity rather than a result of cell damage, thereby reinforcing the therapeutic potential of these peptides.

## 3. Materials and Methods

### 3.1. Materials and Reagents

Dried BM, processed according to the Pharmacopoeia of the People’s Republic of China, was purchased from Yao Ding Agricultural Development Co., Ltd. (Jingmen, China). All samples were preserved at −80 °C before being used in the experiments. HUVEC cells were purchased from Bena Bioengineering Company (Wuhan, China). The following key enzymes for SGD were purchased from Shanghai Macklin Biochemical Co., Ltd. (Shanghai, China): α-amylase (EC 3.2.1.1), pepsin (EC 3.4.23.1), and trypsin (EC 3.4.21.4). Additionally, ACE and the reaction substrate N-[3-(2-furylacryloyl)]-L-phenylalanyl-glycyl-glycine (FAPGG) were purchased from Sigma-Aldrich (Shanghai, China). Other chemical reagents, such as NaOH and HCl, were purchased from Beijing Solarbio Science & Technology Co., Ltd. (Beijing, China).

### 3.2. In Vitro Digestion Process of BM

SGD was performed following the static in vitro digestion protocol outlined in the INFOGEST 2.0 [29,30], with minor modifications. Pulverized BM was homogenized with ultrapure water (1:2, *w*/*v*) prior to digestion.

#### 3.2.1. Oral Phase

Three aliquots of the sample solution (5 mL,0.5 mg/mL) were prepared. Each aliquot was mixed with 3.5 mL of simulated salivary fluid (SSF), 0.5 mL of α-amylase (1500 U/mL), 25 μL of 0.3 M CaCl_2_, and 0.975 mL of ultrapure water. The mixture was incubated at 37 °C for 5 min to obtain the oral hydrolysate (BMHs-O).

#### 3.2.2. Gastric Phase

Two aliquots of the BMHs-O were subjected to gastric digestion. The oral bolus was mixed with 7.5 mL of simulated gastric fluid (SGF), 1.6 mL of pepsin (25,000 U/mL), 5 µL of 0.3M CaCl_2_, and 0.895 mL of ultrapure water. The pH was adjusted to 2.5 using 1M HCl. The mixture was incubated at 37 °C for 2 h to yield the gastric hydrolysate (BMHs-G).

#### 3.2.3. Intestinal Phase

One aliquot of the BMHs-G was further processed for intestinal digestion. The gastric chyme was mixed with 11 mL of simulated intestinal fluid (SIF), 5 mL of pancreatin 800 U/mL, 40 µL of 0.3 M CaCl_2_, and 3.96 mL of ultrapure water. The pH was adjusted to 7.0 using 1 M NaOH, followed by incubation at 37 °C.

SSF, SGF, and SIF were prepared fresh prior to use; specific compositions are detailed in Table 3. Hydrolysates from each stage (BMHs-O, BMHs-G, BMHs-I) were centrifuged at 7000 rpm for 15 min at 4 °C. The supernatants were collected, freeze-dried, and stored at −40 °C.

### 3.3. Analysis of MW Distribution and Amino Acid Content

The MW distribution of BMHs from the respective stages of SGD was determined using an established method with minor adjustments [20]. The hydrolysates were dissolved in a 0.15 mol/L phosphate-buffered (PB) solution (pH = 7.0) at 1.00 mg/mL, and the resulting solution was clarified by passage. A High-Performance Liquid Chromatography (HPLC) system was employed for the analysis with a TSKgel G2000PWxL column (7.8 × 300 mm).

For amino acid composition analysis, phenylisothiocyanate (PITC) pre-column derivatization was used for amino acid analysis [31]. The BMHs samples (8 mg) were determined following acid hydrolysis in 6 M HCl containing 0.1% phenol at 110 °C for 24 h. The resulting hydrolysates were dried via rotary evaporation (70 °C), reconstituted in ultrapure water, and then derivatized for 1 h using phenylisothiocyanate (PITC) in a triethylamine-acetonitrile solution. The PITC derivatives were subsequently purified by n-hexane extraction; the lower aqueous phase was collected, diluted, and filtered (0.22 µm) prior to analysis. Derivatized amino acids were analyzed using HPLC with a Diamonsil^®^ AAA analytical column and ultraviolet detection at 254 nm. Separation was achieved under gradient conditions with a constant flow rate of 0.6 mL/min. The mobile phase included solution A mobile phase A (sodium acetate buffer, pH 6.5) and mobile phase B, a methanol-acetonitrile-water solution (20:60:20, by volume). The assignment of each amino acid peak was achieved by aligning its retention time with that of a known standard, while its percentage composition was calculated from the integrated peak area.

### 3.4. Determination of ACE Inhibitory Effect

The ACE inhibitory rate was assessed using a previously reported protocol [32,33] with minor adjustments. Using 50 mM borate-buffered 4-(2-hydroxyethyl)-1-piperazineethanesulfonic acid (HEPES) (pH 8.3) as the solvent for all reagents, the assay was performed in 96-well plates. Specifically, 100 µL of each sample (1mg/mL), 50 µL of ACE (0.1 U/mL), and 50 µL of substrate FAPGG (1 mM) were added to the wells. The negative control wells received 100 µL of the buffer solution in place of the sample, while Captopril served as the positive control. Measurements were conducted using a two-point endpoint method. The absorbance at 340 nm was recorded immediately after mixing (a1, b1) and again after incubating at 37 °C for 30 min (a_2_, b_2_).

(1) $$ACE\;inhibition\;rate=\frac{A-B}{A}\times 100\%$$

where A = a_1_ − a_2,_ and B = b_1_ − b_2_. The initial absorbance of the blank: a_1_—Initial absorbance of the blank group (no inhibitor); b_1_—Initial absorbance of the sample group (with potential inhibitor); a_2_: Absorbance of the blank group post-incubation; b_2_: Absorbance of the sample group post-incubation.

### 3.5. BMHs-I Stability Analysis

The effects of temperature, pH, metal ions and NaCl were analyzed.

#### 3.5.1. Stability of NaCl Concentration

Peptide solutions (1 mg/mL) were prepared directly in NaCl solutions with varying concentrations (0.2, 0.4, 0.6, 0.8, and 1.0 mol/L). The mixtures were incubated for 2 h, and centrifuged at 5000 rpm for 10 min. The supernatants were collected and evaluated for antihypertensive activity. A peptide solution without NaCl was used as the control.

#### 3.5.2. Evaluation of pH Stability

1 mg/mL solution of BMHs-I was prepared. The solution was then titrated to pH values of 2, 4, 6, 8, and 10 with 1 M NaOH or 1M HCl and incubated for 2 h. The samples were centrifuged at 5000 rpm for 10 min. Prior to analysis, the pH of the supernatants was returned to 8.3 for the determination of the ACE inhibition rate. The untreated peptide solution (pH 8.3) served as the control.

#### 3.5.3. Investigation of Thermal Stability

Peptide solutions (1 mg/mL) of varying molecular weights were incubated in a water bath at 20, 40, 60, 80 and 100 °C for 2 h. Subsequently, samples were cooled to 37 °C and centrifuged at 5000 rpm for 10 min. The ACE inhibitory activity of the collected supernatants was measured, using a solution kept at 37 °C as the control.

#### 3.5.4. Stability of Metal Ions

Prepare peptide solution (1 mg/mL) in 100 μg/mL MgCl_2_, ZnCl_2_, CuCl_2_, and CaCl_2_ solutions, respectively. After incubating at 37 °C for 2 h, the samples were centrifuged at 5000 rpm for 10 min. The ACE inhibitory activity of the supernatant was determined. A peptide solution without metal ions was used as the control.

### 3.6. Purification of Peptides in the BMHs-I

BMHs-I were dissolved at 2 mg/mL (10 mL) and filtered through a 0.45 µm membrane. The supernatant was first added to the 10 kDa filter unit and centrifuged at 4000× *g* for 40 min at 4 °C. The retentate (molecular weight > 10 kDa) was collected as fraction BMHs-IU1. The permeate (molecular weight < 10 kDa) was subsequently loaded onto the 5 kDa filter unit and centrifuged under the same conditions. The resulting retentate (5–10 kDa) was collected as fraction BMHs-IU2, while the final permeate (<5 kDa) was collected as fraction BMHs-IU. All fractions were lyophilized and stored at −40 °C for further analysis 3.4, and the components with the highest activity were selected for further study.

### 3.7. Identification of Peptides by Liquid Chromatograph Mass Spectrometer

#### 3.7.1. Sample Preparation

The sample was ground into a fine powder using liquid nitrogen and extracted with a lysis buffer containing 8 M urea, 100 mM dithiothreitol (DTT), 4% 3-[(3-cholamidopropyl) dimethylammonio]-1-propanesulfonate (CHAPS), and protease inhibitors. The resulting suspension underwent ultrasonication on ice for 2 min. Following centrifugation at 12,000× *g*, the supernatant was loaded into 10 kDa molecular weight cut-off (MWCO) spin units and centrifuged at 12,000× *g*. To optimize peptide recovery, the filters were washed twice with 0.25% acetic acid, involving centrifugation at 12,000× *g* for 20 min per step. The flow-through fraction containing peptides was harvested, freeze-dried, and resuspended in 0.1% trifluoroacetic acid. Finally, the samples were desalted via C18 solid-phase extraction cartridges before LC-MS assessment.

#### 3.7.2. LC-MS/MS Analysis

Peptides were fractionated using a Vanquish Neo UHPLC system (Thermo Scientific, Waltham, MA, USA) equipped with mobile phases of 0.1% formic acid in water (Buffer A) and 0.1% formic acid in 80% acetonitrile (Buffer B). Upon equilibrating with 96% Buffer A, samples were loaded onto a PepMap Neo trap column (C18, 5 µm, 300 µm × 5 mm) and resolved via a μPAC Neo High Throughput analytical column.

The 15 min elution gradient was programmed as follows:

0–0.1 min, 4–8% B

0.1–1.2 min, 8–10% B

1.2–11.2 min, 10–28% B

11.2–12.5 min, 28–45% B

12.5–13.5 min, 45–99% B

13.5–15 min, 99% B

Mass spectrometry was conducted on an Orbitrap Astral instrument operating in positive ion Data-Dependent Acquisition (DDA) mode.

MS_1_ settings were spray voltage, 2.2 kV; scan range, 380–980 *m*/*z*; resolution, 240,000; automatic gain control (AGC) target, 500%; maximum IT, 3 ms.

MS_2_ parameters included: higher-energy collisional dissociation HCD activation with 25% normalized collision energy; resolution, 80,000; AGC target, 500%; maximum IT, 3 ms; RF-lens, 40%; isolation window, 2 Th; cycle time, 0.6 s.

#### 3.7.3. Database Search

The resulting data were processed using PEAK Studio (De Novo mode) [34]. The search parameters for PEAK Studio included an unspecific enzyme setting, with mass tolerances of 10 ppm for precursor ions and 0.02 Da for-fragment ions. No fixed modifications were applied, while variable modifications accounted for oxidation (M) and acetylation (Protein N-terminus). To ensure high-confidence identifications, false discovery rate (FDR) thresholds were set at ≤1% for both peptide-spectrum matches (PSMs) and protein identifications. Additionally, only peptides with a De Novo score ≥ 70% were retained, and a maximum of 2 variable post-translational modifications (PTMs) per peptide were allowed.

### 3.8. In Silico Screening for ACEIPs

Peptides were filtered using an ALC threshold of >90% [35]. Bioactivity was predicted via Peptide Ranker (score > 0.9) (https://distilldeep.ucd.ie/PeptideRanker/, accessed on 14 October 2025). Candidates were subsequently restricted to sequences < 10 residues containing C-terminal aromatic and basic amino acids (W, F, R, K, L) [36]. Finally, toxicity and allergenicity were assessed using ToxinPred (https://crdd.osdd.net/raghava/toxinpred/index.html, accessed on 14 October 2025) and AllerTOP v.2.0 (https://www.ddg-pharmfac.net/allertop_test/, accessed on 14 October 2025) platforms, respectively; only peptides predicted to be non-toxic and non-allergenic were selected.

### 3.9. Molecular Docking Simulation of Putative Bioactive Peptides to ACE

Molecular docking simulations employing AutoDock Vina (v. 1.1.2) were performed to characterize the putative interactions and calculate the binding energies between the identified peptides and the ACE active site. This analysis aimed to elucidate the principal intermolecular forces contributing to the stability of the peptide-enzyme complex. The crystal structure of ACE (PDB ID: 1O8A) was obtained from the RCSB PDB database (http://www1.rcsb.org/, accessed on 14 October 2025). Peptide structures were modeled using ChemDraw 23.1.1 software. The docking grid for 1O8A was centered at coordinates 39.706 × 40.313 × 39.701 with grid dimensions set to 152 × 186 × 162 along the XYZ axes. Nine independent docking iterations were performed for each ligand. Synthesis and activity verification of peptides.

### 3.10. Synthesis and Activity Assessment of Peptides

Peptides with a purity of >95% (confirmed by LC-MS) were commercially synthesized by SCI-GO (Hefei, China), stored at −80 °C, and subsequently assessed for ACE inhibitory activity following the procedure in Section 3.4.

### 3.11. Determination of Inhibition Mode by Kinetic Analysis

To characterize the inhibitory kinetics of the peptides against ACE, an established protocol was adopted and slightly modified [37]. The inhibitory kinetics of the peptides against ACE were determined as described in detail in Section 3.4, with modifications to peptide concentrations (Control, 25 and 50 µM) and FAPGG substrate concentrations (0.25, 0.5, 1, 2, 4, and 8 mM). The inhibition mode was confirmed using Lineweaver-Burk plots, and the kinetic parameters (Km and Vmax) were determined based on the Michaelis-Menten model.

### 3.12. Cell Culture

HUVECs were maintained in high-glucose Dulbecco’s Modified Eagle Medium (DMEM) containing 10% fetal bovine serum and antibiotics at 37 °C. For the experiments, cells were subjected to treatment with synthetic peptides at concentrations ranging from 3.125 to 200 µM.

### 3.13. Cell Viability Analysis

HUVECs were plated in 96-well plates at a density of 3 × 10^5^ cells/mL and cultured for 24 h at 37 °C. After a 4 h incubation with 100 µL of cell counting kit-8 solution per well, the absorbance at 450 nm was quantified using a microplate reader.

### 3.14. Statistical Analysis

All experiments were performed in triplicate (*n* = 3), except for the cellular assays, which were conducted in five replicates (*n* = 6). Data was analyzed by one-way ANOVA with Duncan’s post hoc test using SPSS (v21.0), with statistical significance set at *p* < 0.05.

## 4. Conclusions

This study aimed to explore the potential of non-venom proteins from BM as a novel source of ACEIPs with blood pressure-regulating bioactivity. By employing a strategy combining simulated gastrointestinal digestion with bioinformatic screening, we successfully identified three novel peptides—PPSPPRW, WGFTKF, and PSLFPPRL. These peptides exhibit significant ACE inhibitory activity, defined safety margins at physiological concentrations, and high stability under diverse temperature and pH conditions. Furthermore, kinetic and molecular docking analyses revealed that these peptides exert their effects through distinct mechanisms: competitive inhibition (PPSPPRW and WGFTKF) and mixed-type inhibition (PSLFPPRL), enriching the theoretical understanding of peptide-enzyme interactions.

On a practical level, this study suggests that BM proteins are a promising high-quality raw material for the development of pharmaceutical lead compounds. However, a limitation of this exploratory study is the reliance on in vitro kinetics and in silico models without direct in vivo pharmacodynamic validation. Consequently, future research should prioritize two key directions by first validating the in vivo antihypertensive efficacy of the lead peptide (PPSPPRW) in hypertensive animal models and second, systematically investigating the metabolic stability and transepithelial transport of these peptides to substantiate their translational potential as therapeutic agents.

In summary, this study provides a scientific basis for the comprehensive utilization of traditional medicinal animal resources and opens new avenues for the development of modern peptide-based therapeutics.

## Figures and Tables

**Table 1:** Amino Acid Composition of BMHs Across Processing Stages.

Amino Acid (%)	BMHs-O	BMHs-G	BMHs-I
Asp	15.15	11.83	10.82
Glu	13.78	19.61	17.33
Ser	12.33	7.63	7.04
His	3.52	2.99	1.81
Arg	5.44	7.73	8.76
Thr *	3.21	3.9	3.32
Ala	8.85	6.14	5.31
Pro	7.45	5.49	5.02
Tyr	3.01	3.97	3.07
Val *	3.80	4.53	4.73
Met *	2.00	1.51	1.62
Ile *	2.35	3.21	3.64
Leu *	4.31	5.48	7.51
Phe *	4.11	3.96	3.96
Lys *	6.24	6.01	5.92
Gly	4.45	6.01	5.53

Note * denotes essential amino acids.

**Table 2:** Physicochemical and safety properties of the selected peptides.

Number	Peptide	Length	Peptide Ranker Score	Binding Energy (Kcal/mol)	Non-Toxin	Non-Allergen
1	FKAAFDMF	8	0.902981	−7.9	yes	no
2	PSLFPPRL	8	0.960754	−9.9	yes	yes
3	HWPWMKL	7	0.973186	−8.4	yes	no
4	PPSPPRW	7	0.965035	−11.1	yes	yes
5	LGGPLRF	7	0.915137	−8.7	yes	no
6	HWPWMK	6	0.975617	−8.6	yes	yes
7	WGFTKF	6	0.961909	−10.4	yes	yes
8	FQWYR	5	0.953664	−10.2	yes	yes

**Table 3:** Composition and parameters of the simulated digestive fluids used in the SGD.

Constituent	SSF		SGF		SIF	
	Volume (mL)	Concentration (mmol/L)	Volume (mL)	Concentration (mmol/L)	Volume (mL)	Concentration (mmol/L)
KCl	15.1	15.1	6.9	6.9	6.8	6.8
KH_2_PO_4_	3.7	3.7	0.9	0.9	0.8	0.8
NaHCO_3_	6.8	13.6	12.5	25	42.5	85
NaCl	-	-	11.8	47.2	9.6	38.4
MgCl_2_(H_2_O)_6_	0.5	0.15	0.4	0.1	1.1	0.33
(NH_4_)_2_CO_3_	0.06	0.06	0.5	0.5	-	-

## Data Availability

All original data from this work are contained in the article. Readers seeking further details may direct their inquiries to the corresponding author upon reasonable request.
